# COVID-19: Implications for People with Chagas Disease

**DOI:** 10.5334/gh.891

**Published:** 2020-10-13

**Authors:** Ezequiel José Zaidel, Colin J. Forsyth, Gabriel Novick, Rachel Marcus, Antonio Luiz P. Ribeiro, Maria-Jesus Pinazo, Carlos A. Morillo, Luis Eduardo Echeverría, Maria Aparecida Shikanai-Yasuda, Pierre Buekens, Pablo Perel, Sheba K. Meymandi, Kate Ralston, Fausto Pinto, Sergio Sosa-Estani

**Affiliations:** 1 Sanatorio Güemes, Buenos Aires, AR; 2 Pharmacology Department, School of Medicine, University of Buenos Aires, AR; 3 Drugs for Neglected Diseases initiative-Latin America, Rio de Janeiro, BR; 4 Swiss Medical Group, Buenos Aires, AR; 5 Friedman School of Nutrition Science and Policy, Tufts University, Boston, US; 6 Latin American Society of Chagas, Washington, DC, US; 7 Internal Medicine Department, School of Medicine, Federal University of Minas Gerais (UFMG), Belo Horizonte, BR; 8 Hospital das Clínicas, UFMG, Belo Horizonte, BR; 9 ISGlobal, Hospital Clínic – University of Barcelona, Barcelona, ES; 10 Libin Cardiovascular Institute, University of Calgary, Calgary, CA; 11 Department of Cardiology, Cardiovascular Foundation of Colombia, Floriblanca, CO; 12 Department of Infectious and Parasitic Diseases, School of Medicine, University of São Paulo, São Paulo, BR; 13 Tulane University School of Public Health and Tropical Medicine, New Orleans, US; 14 World Heart Federation, Geneva, CH; 15 Department of Non-communicable Disease Epidemiology, London School of Hygiene and Tropical Medicine, London, UK; 16 Center of Excellence for Chagas Disease at Olive View-UCLA Medical Center, Sylmar, US; 17 Department of Cardiology, CCUL, Lisbon School of Medicine, Universidade de Lisboa, PT; 18 Epidemiology and Public Health Research Center, (CIESP-CONICET), Buenos Aires, AR

**Keywords:** COVID-19, Chagas Disease, Neglected Tropical Disease

## Abstract

As the global COVID-19 pandemic advances, it increasingly impacts those vulnerable populations who already bear a heavy burden of neglected tropical disease. Chagas disease (CD), a neglected parasitic infection, is of particular concern because of its potential to cause cardiac, gastrointestinal, and other complications which could increase susceptibility to COVID-19. The over one million people worldwide with chronic Chagas cardiomyopathy require special consideration because of COVID-19’s potential impact on the heart, yet the pandemic also affects treatment provision to people with acute or chronic indeterminate CD. In this document, a follow-up to the WHF-IASC Roadmap on CD, we assess the implications of coinfection with SARS-CoV-2 and *Trypanosoma cruzi*, the etiological agent of CD. Based on the limited evidence available, we provide preliminary guidance for testing, treatment, and management of patients affected by both diseases, while highlighting emerging healthcare access challenges and future research needs.

## Introduction

In 2020, the SARS-CoV-2 virus, which causes COVID-19, took the world by storm. The biological and social implications of this global pandemic may not be fully understood for years. However, what is increasingly clear is that like other diseases, COVID-19 disproportionately affects those living at the social margins, while also being particularly severe in older individuals and those with certain underlying health conditions. These are both key considerations as COVID-19 increasingly intersects with the world’s neglected diseases, including Chagas disease (CD), a multi-systemic disorder caused by *Trypanosoma cruzi* (*T. cruzi*) that can affect the cardiovascular, digestive and central nervous systems [1]. CD is endemic in much of Latin America, which is increasingly bearing the brunt of the pandemic. The first reported Latin American case of COVID-19 was on February 26, 2020 in Brazil; by September 4th, there were over 7.5 million confirmed infections and over 280,000 deaths in the region [2], with numbers still increasing rapidly at the time of writing this paper. Several aspects of CD are of particular concern in light of what we know about COVID-19: Many people living with CD are socioeconomically vulnerable and have limited access to healthcare, the vast majority are undiagnosed, most are aging, and over a million have already progressed to a cardiac form of the disease [3,4]. This paper will focus on the potential interactions between CD and COVID-19 in coinfected individuals, which become increasingly important as the pandemic spreads rapidly through Latin American countries where CD is endemic.

On March 30, 2020 the Inter-American Society of Cardiology and the World Heart Federation published a roadmap that provides a comprehensive overview of CD, with steps for improving healthcare access [5]. Also in March of 2020, the Chagas Coalition prepared a question and answer document with information about COVID-19 for people with CD.^1^ In April 2020, the Drugs for Neglected Diseases initiative’s Chagas Research Platform reconvened some members from the roadmap writing group as well as other experts, including several members of the Chagas Coalition, to write a follow-up paper exploring how COVID-19 might impact people living with CD, and to provide preliminary guidance based on the limited amount of evidence available on the topic. The group consisted of several CD experts, some of whom have been on the front lines during the current crisis treating patients with COVID-19 or performing research on new treatments. The following document summarizes the consensus opinion of these experts on SARS-CoV-2/*T. cruzi* coinfection.

## Pathophysiology of COVID-19 Infection in Relation to that of CD

CD is mainly transmitted through various species of hematophagous insects, although it can also be transmitted transplacentally, through infected blood transfusions or organ donations, laboratory accidents, needle sharing among intravenous drug users (IVDU), and orally through food and drink contaminated with triatomines, their feces, or secretions from some host reservoir species. After infection and an incubation period of between 15 and 40 days, the acute phase of the disease generally lasts for one to two months and is followed by an indeterminate phase, when no clinical manifestations are observed. After decades in this silent state, roughly one-third of patients develop a chronic form of the disease characterized by organ damage, mainly to the cardiovascular (CV) and gastrointestinal (GI) systems. The most serious sequelae of CD are stroke, sudden death from brady- or tachyarrhythmias, and congestive heart failure [1,6].

COVID-19 interacts with the CV system on multiple levels. SARS-CoV-2 binds to the human angiotensin-converting enzyme 2 (ACE2) receptor mainly expressed in the lungs, heart, and vascular endothelium. Although analysis of the precise consequences is in its infancy, this interaction may trigger an inflammatory response that, in turn, may lead to increasing myocardial injury and dysfunction [7,8]. It is uncertain whether the altered immune state characteristic of COVID-19 disease can act as a potential trigger for CD progression, and how this might be influenced by both certain parasitic factors (type of strains, load of parasites) as well as host factors (genetic susceptibility and immune state, specifically IFN-Ɣ axis).

While parasitemia is low-level and evanescent in chronic CD, pharmacologic and disease-induced immunosuppression risk reactivation of parasitemia [9,10]; therefore, there is a concern that COVID-19 disease could potentially trigger reactivation of CD. This potential reactivation could be caused by an acquired hemophagocytic lymphohistiocytosis-like disease (cytokine storm), the virus itself, or even the use of some COVID-19 treatments such as steroids, hydroxychloroquine [5] and other immune-modulating drugs (i.e. tocilizumab or other interleukin inhibitors), as interleukins are related to the progression of CD [11,12]. This may be influenced by certain parasitic factors or host factors.

### Implications for chronic Chagas cardiomyopathy

The pathogenesis of chronic Chagas cardiomyopathy (CCC) involves a complex interaction between different processes related to tissue damage due to parasite persistence, inflammation, specific immune response, fibrosis, dysautonomia, and microvascular changes [13]. Chronic, persistent infection of the myocardium elicits an inflammatory response which, although necessary for the control of parasite proliferation, results in tissue damage leading to myocardial fibrosis and cardiac remodeling [14]. The pro-inflammatory response includes, but is not limited to, secretion of Th1 cytokines and chemokines, eicosanoids, and endothelin-1 [14].

Similar to the case for *T. cruzi* infection, direct damage to cardiac tissue also is possible with SARS-CoV-2, which binds to the ACE2 receptor to enter type 2 pneumocytes, macrophages, perivascular pericytes, and cardiomyocytes. This may lead to myocardial dysfunction and damage, endothelial dysfunction, microvascular dysfunction, plaque instability, and myocardial infarction (MI) [15]. Initial immune and inflammatory responses induce a severe cytokine storm [16], including cytokines and chemokines frequently related to the inflammatory response implicated in the pathogenesis of CCC, such as interleukin (IL)-6, TNF-alpha, and CXCL10 [17,18]. Indeed, COVID-19 related myocarditis cases have been reported and are thought to be a combination of direct viral injury and cardiac damage due to the host immune response [19].

Further depression of the ventricular function by COVID-19 could be caused by additional mechanisms, such as myocardial infarction and microvascular dysfunction, also found in *T. cruzi* infection [15]. Furthermore, arrhythmia is recognized as one of the possible clinical manifestations of COVID-19 patients, thus COVID-19 could plausibly precipitate arrhythmias in patients with an arrhythmogenic substrate, such as CCC [19].

Finally, COVID-19 may predispose patients to thrombotic disease, both in the venous and arterial circulations, due to excessive inflammation, platelet activation, endothelial dysfunction, and stasis [20]. There are reports of upregulated procoagulative activity in the plasma of chronic CD patients [21] and thromboembolic manifestations are also more frequent in these patients with CCC [22], although it is not clear if this interaction has clinical relevance.

### Implications for chronic indeterminate CD

While patients living with the indeterminate form of CD are usually outpatients without manifest symptoms, they still require ongoing surveillance and care, which has become increasingly difficult due to the pandemic. For instance, patients with chronic indeterminate CD need yearly follow-up for cardiac tests, as 2–5% progress to chronic symptomatic disease annually, and ultimately 30–40% develop cardiac or GI complications [1]. They may also benefit from antiparasitic treatment, which, in the case of women of childbearing age, can interrupt congenital transmission [23,24]. However, the 2-month course of treatment requires ongoing monitoring and laboratory testing due to the potential for side effects. This essential care for patients with chronic indeterminate CD is likely to be hampered by the pandemic, both because of the potential risk of SARS-CoV-2 infection from attending healthcare facilities for routine appointments (or the need to use public transportation to travel to and from appointments), and because of postponements or delays in routine care as healthcare personnel and resources are focused on COVID-19 cases.

Furthermore, if assessed with more sensitive technology, including echocardiography, Holter, and magnetic resonance with late gadolinium enhancement, a small number of indeterminate-phase patients may be reclassified as having CCC due to the presence of areas of fibrosis with wall motion abnormalities [25,26,27,28,29,30]. Therefore, an unknown proportion of individuals classified as being in the indeterminate phase may indeed develop arrhythmias or other cardiovascular complications if challenged with a cytokine storm such as that triggered by COVID-19.

### Implications for gastrointestinal and neurological forms of CD

Atypical chest pain, abdominal pain, and nausea are nonspecific symptoms related to upper digestive CD involvement, and all three have been related to COVID-19 pulmonary and extrapulmonary clinical presentation [31,32]. However, constipation is the main symptom of lower digestive tract involvement due to *T. cruzi* infection which is the opposite of diarrhea, the main GI symptom observed in extrapulmonary forms of COVID-19. The presence of GI symptoms, widely described in COVID-19 case series and present in 3–11.6% of patients with COVID-19 [31], has been associated with high ACE2 expression in the GI tract that could indicate the potential of virus mutation towards increased transmissibility, decreased virulence, and multiorgan infection [31]. When GI symptoms that occur in both diseases are noted in an individual in an area of high SARS-CoV-2 transmission, etiological consideration must be given to either CD progression or COVID-19, considering the implications in terms of mutation and transmissibility.

There is increasing evidence that coronaviruses are associated with neurological disorders [33]. Studies on severe acute respiratory syndrome (SARS) and the Middle East respiratory syndrome (MERS) suggest that coronaviruses are neurotropic. A systematic review of the literature until April 2020 associated multiple neurological disorders with COVID-19, including encephalitis, demyelination, and neuropathy [34]. COVID-19 could potentially induce the development of chagasic neuropathy, which is sometimes observed in chronic CD.

### Congenital cases

Maternal-fetal transmission of *T. cruzi* occurs in an average of 5% of pregnancies of mothers with chronic CD [35]. Parasite load is a key determinant of congenital transmission [36]. The immunological response to COVID-19 is extensive, and its impact on *T. cruzi* parasitic load is unknown. Should it be found that COVID-19 increased parasitemia in pregnant women with CD, it could increase the likelihood of maternal-fetal transmission. Screening of infants born to *T. cruzi*-infected mothers remains crucial and could potentially be disrupted by the negative impact of COVID-19 on access to care [37,38].

### Immunosuppressed patients

Immunosuppressed patients are at increased risk of severe COVID-19, especially those with aggressive underlying disease, active immunosuppressive treatment, or lymphopenia. Overproduction of cytokines during COVID-19 infection leads to significant tissular damage, particularly in the lungs. This intense COVID-19 inflammatory process in immunosuppressed CD patients could influence the evolution of disease and potentially trigger CD reactivation, due either to viral infection interference, such as seen in HIV infection [39], or to possible immunosuppressive therapy for COVID-19 [9,10] and is associated with the severity of underlying diseases. Conversely, any approach that improves the immune response at this level is desirable, either using antiviral or cytokine blocking agents (IL-6, IL-1β, TNF-a) [40]. On the other hand, there is the risk of inducing clinical activation of autoimmune disease in individuals with asymptomatic COVID-19 and, consequently, a potential reactivation of CD [41].

## Epidemiological Considerations

The spread of the COVID-19 pandemic in countries affected by CD raises concerns for several reasons. The population with *T. cruzi* infection, which numbers over six million people worldwide [3,42], is aging, at risk of CCC, has a significant burden of comorbidities, and is socioeconomically vulnerable. All these factors could potentially increase the impact of COVID-19 in this population, especially within a scenario of weakened and overloaded health systems.

In most countries where CD transmission has been controlled or reduced, CD patients are becoming older and suffer from comorbid conditions [43]. Further investigation is needed to fully understand the impact of comorbid illnesses in CD patients, including the role of immunosuppressive conditions arising from CD or from CD therapies. In addition to age, many chronic medical conditions, such as diabetes, COPD and other cardiovascular diseases are also known risk factors for COVID-19 mortality. Moreover, elderly CCC patients have higher risk of death than age-matched seronegative individuals [43]. It is likely, therefore, that CCC, in part because of its association with age and other chronic conditions, along with the challenging socioeconomic context affecting many people with CD, would further increase the risks of severe COVID-19 infection.

Although the incidence of new CD infections is around 30,000 annually [3], this number has declined in recent decades, meaning a high proportion of people living with CD are older or aging [44]. While >80% of COVID-19 cases are mild or asymptomatic, severe cases are more common among older adults. In a retrospective study, the risk of mortality increased by 1.03–1.17% with every year of increased age in patients from Wuhan [45]. Another study using data from various countries estimated a case fatality ratio of 4.5% in individuals older than 60, compared to 1.4% in those younger than 60, with the highest rates in patients over 80 [46]. In the United States, 78.6% of deaths have occurred in people 65 or older [47].

Over one million people in the Americas suffer from CCC [3], and underlying cardiovascular disease is a major risk factor for hospitalization and death from COVID-19. The incidence of acute cardiac injury from COVID-19 has been reported as 8% in hospitalized patients (48), and (in one Chinese study) was much more prevalent in deceased (59%) than recovering patients (1%) [49]. Cardiovascular disease was also identified in 30% of COVID-19 related deaths in Italy [50], while age > 60 and Charlson Comorbidity Index > 3 were associated with greater mortality in a U.S. cohort of 1305 hospitalized patients [51].

Finally, several studies have noted high levels of comorbidities among both patients with CD and those with severe forms of COVID-19. One Brazilian study identified a mean of 2.7 chronic comorbidities in CD patients [52]. In 168 CD patients in São Paulo, 51.2% had hypertension and 23.8% had diabetes mellitus [53]. Another study in a younger sample of 137 patients in Switzerland found 2.9% had diabetes and 17% hypertension [54]. Both hypertension and diabetes were associated with high mortality rates from COVID-19 in China (7.3% and 6.0% respectively) [55], and were 2–3 times more prevalent in severe vs. non-severe hospitalizations [48]. Diabetes was also prevalent in a third of deaths in an analysis of Italian COVID-19 data [56]. It is important to note that these comorbidities also reflect the older age of the populations which are especially impacted by both CD and COVID-19.

## Social Context and Access to Healthcare

As the pandemic progresses from Europe and the United States into the Global South, it is increasingly impacting vulnerable populations. In the U.S., COVID-19 has thus far had a greater impact on Blacks and Latinos. According to the CDC, these groups have a higher prevalence of infection and higher weighted distribution of deaths than expected from their distribution in the general population [57]. A higher rate of deaths among people in Brazil who self-identify as black has also been reported [58,59]. These racial disparities are, in turn, framed by historically rooted socioeconomic considerations which often determine who is able to self-isolate and avoid exposure.

Moreover, social vulnerability has long been documented in people with CD [60], and groups with high burdens of CD, including indigenous people, the rural poor, and migrants may face particular challenges in accessing healthcare. People living at or near the poverty level are also especially vulnerable to the economic impact of the pandemic, as experts have warned that years of gains in reducing poverty are now in jeopardy. The UN predicts that a global GDP shrinkage of 3.2% in 2020, will push 34 million more people into extreme poverty [61]. The worsening economic situation threatens to make access to healthcare even more precarious for people living with CD, who are often forced to make difficult decisions between spending time and money on healthcare or on other pressing priorities. Many work in the informal sector or in positions that do not offer paid time off or health insurance.

People with CD may feel more reluctant to seek care in a clinic for fear of exposure to COVID-19, and may, therefore, put off addressing complications related to CD. Indeed, there have been reports of low utilization of services and delayed presentation for chronic conditions since the onset of the pandemic [62,63]. Finally, CD can create a significant emotional burden for affected people who worry about the progression of the disease, and the pandemic may make it harder to access both mental health services and traditional support networks of families and friends. Patients with CD who acquire COVID-19 may experience particular concern and anxiety. As COVID-19 becomes a part of daily reality, creative responses will be needed from patient groups, social workers, mental health practitioners, and others to continue providing support to people with CD.

## Recommendations for Healthcare Providers

### Screening/testing of Chagas disease during the pandemic

While the indications for screening and diagnosing CD are unchanged during the COVID-19 pandemic, the timing of testing depends on the degree to which the diagnosis of CD will affect short term management of the individual being tested. Urgent testing remains appropriate for pregnant women, infants born to seropositive mothers, and any individual about to receive immunosuppression. Screening of blood donations also remains critical. Patients presenting with clinical syndromes suggestive of CD should also receive urgent testing to guide evaluation and therapy. Pregnant women from CD-endemic countries should also be screened to evaluate for the possibility of maternal-fetal transmission. Depending on the circulation of SARS-CoV-2 in the community and local guidelines, widespread screening of individuals without symptoms from endemic countries could be postponed until it can be performed safely, with the understanding that while antiparasitic therapy may be indicated, it can generally be delayed until the patient can report for lab testing and follow-up visits without significant risk of exposure.

### Management of patients coinfected with Chagas disease and COVID-19

Below, preliminary recommendations are provided for patients with CD who acquire COVID-19. The recommendations are divided depending on the patient’s form of CD.

#### Acute forms of CD

Acute cases of CD following congenital, vector, or oral transmission, as well as those via transfusion, laboratory accidents, and other routes generally warrant antiparasitic treatment as early as possible, even in the context of the COVID-19 pandemic. If the patient is coinfected with COVID-19 and the acute form of CD, timely antiparasitic treatment of CD is needed, but clinicians should be mindful of the severity of COVID-19 symptoms.

##### Congenital CD

Cases of congenital transmission are acute cases of CD, and treatment is effective with few side effects. COVID-19 is generally not severe in infants and children [64]. If the child does not have COVID-19 symptoms, antiparasitic treatment should be given as soon as the diagnosis of *T. cruzi* infection is established [37].

##### Reactivation

Patients must be admitted to the hospital and receive antiparasitic treatment with benznidazole for 60 days. However, if a full 60 days of treatment is not possible because of adverse events, treatment should still be continued for as close to 60 days as possible using anti-histaminic and/or anti-inflammatory drugs. Reactivation involving myocarditis/meningoencephalitis is of particular concern due to the occurrence of vascular/nervous system/myocardium involvement by COVID-19 and should be carefully monitored in an intensive care unit.

##### Immunosuppression without reactivation

In this case, antiparasitic treatment could be delayed until the patient can safely attend a clinic, depending on self-isolation guidelines and the risk of exposure to COVID-19. However, CD reactivation with signs in target organs should be closely monitored by clinical follow-up, direct microscopy (concentration methods) on peripheral blood and/or secretions [39], and, if possible, quantitative PCR during COVID-19 infection according to local protocols.

#### Indeterminate chronic CD

From an individual health management point of view, people with asymptomatic infection (in the indeterminate clinical form of CD) are those in whom there is evidence of *T. cruzi* infection, but no evidence of organ damage (mainly cardiovascular or digestive), assessed by non-specific symptoms and low sensitivity tests to detect early organ damage, such as electrocardiogram, chest X-ray, and barium swallow and enema [65]. With or without antiparasitic treatment, regular monitoring is recommended to evaluate the clinical condition during follow-up and to quickly detect treatment failure and/or clinical progression.

As a general recommendation, and given the current epidemiological situation, it is important to assess the risk-benefit of referring a patient to a healthcare center. It is essential to preserve patient safety in terms of preventing new cases of COVID-19. However, it is also important from a public health perspective to optimize existing resources for health care. Outpatient visits as well as regular cardiovascular and gastrointestinal tests could be delayed if CD patients are stable. The use of telehealth tools for virtual consultation is highly recommended, including incorporating advice to patients about the need to contact healthcare facilities in case of the onset of symptoms, either from CD or possible COVID-19 infection.

### Etiological treatment of Chagas disease

Etiological (antiparasitic) treatment in patients with CD without evidence of organ involvement is recommended for acute cases and most chronic cases in the indeterminate form or with only mild cardiomyopathy [5,66]. In the current context of the pandemic, however, two main aspects should be considered: 1) drug characteristics and potential interactions with current treatment of COVID-19, and 2) the urgency of the indication for anti-*T. cruzi* drugs.

#### Drug characteristics

Benznidazole (BZN) and nifurtimox (NFX) are the two drugs accepted by regulatory agencies for antiparasitic treatment of CD. BZN (N-benzyl-2-nitro-1-imidazole acetamide) is a nitroimidazole that inhibits DNA, RNA, and protein synthesis of *T. cruzi*. NFX (5-nitrofuran 3-methyl-4-(5’-nitrofurfurylideneamine) tetrahydro-4H-1,4-tiazine-1,1-dioxide) is a nitrofuran derivative whose mechanism of action involves various reduction and oxidation reactions of its nitro constituent, leading to the production by parasite enzymes of a variety of reactive oxygen species that react with cellular macromolecules and are lethal to the parasite. NFX also leads to the inactivation of a critical trypanosomal enzyme, trypanothione reductase [67].

In both cases, the mechanism of action is not completely described, and exploration of the potential interactions between BZN and NFX with most common drugs used in COVID-19 management should be taken into consideration and further explored. Due to the hepatic metabolism of BZN (95%) and NFX (>99%), hepatotoxicity in combination with anti-COVID-19 drugs must be monitored.

#### Treatment indications and follow-up of patients under treatment

Etiological treatment of *T. cruzi* infection is an emergency only under very specific circumstances [68]. Even if adverse drug reactions (ADRs) related to BZN and NFX are non-severe in most cases [69], close follow-up of patients who start a BZN or NFX regime is recommended to identify side effects promptly and to monitor hepatic and hematologic function [54,70,71]. During the pandemic, delaying initiation of etiological treatment regimens for chronic *T. cruzi* infection without organ involvement is a valid course of action in healthcare settings, to avoid unnecessary exposure to COVID-19 and because of limitations in follow-up due to decreased in-person care. Nevertheless, patients diagnosed with COVID-19 may receive immunosuppressive therapy, and close monitoring to diagnose CD reactivation early in its course is recommended. In the case of clinical and/or parasitological evidence of reactivation, starting treatment with BZN or NFX is considered an emergency [68].

If a patient is already receiving BZN or NFX, treatment should be continued, and self-quarantine measures to avoid COVID-19 should be taken. Telehealth tools for treatment follow-up are recommended as well as minimization of contact with healthcare facilities, which would primarily only be indicated to perform laboratory tests usually recommended during treatment to monitor hepatotoxicity and hemogram alterations due to BZN or NFX, or in the event of concerning ADRs. If a patient under treatment with BZN or NFX develops symptomatic COVID-19 infection providers could consider on a case-by-case basis whether to interrupt treatment, depending on the severity of symptoms and the type of treatment required. There is no evidence of drug-drug interaction between antiparasitics for CD and the drugs currently under investigation to treat COVID-19; treatment of CD in the indeterminate form is non-urgent.

Table 1 summarizes guidance for providing etiological treatment of CD during the pandemic, taking into account both the patient’s form of CD and their COVID-19 status.

**Table 1:** Etiological treatment recommendations for Chagas disease in the context of COVID-19 coinfection.*

Chagas disease status	COVID-19 status	Guidance for etiological treatment with benznidazole or nifurtimox

Chronic, indeterminate	Negative	Consider delaying treatment to minimize risk of COVID-19 exposure based on local epidemiological context and current physical distancing regulations.
Chronic, indeterminate	Positive, with or without symptoms	Consider delaying treatment until COVID-19 is completely resolved and based on local epidemiological context and current physical distancing regulations.
Acute cases	Negative or positive, with or without symptoms	Initiate treatment.
Clinical and/or parasitological evidence of reactivation	Negative or positive, with or without symptoms	Initiate treatment.
Chronic, indeterminate, currently undergoing etiological treatment	Positive, symptomatic	Postpone treatment; if immunosuppressive drugs are prescribed in the context of COVID-19 management, closely monitor for reactivation of *T. cruzi* infection by direct microscopy on peripheral blood or fluids and/or quantitative PCR (if available). If reactivation is evident, restart benznidazole/nifurtimox treatment.
Chronic, indeterminate, currently undergoing etiological treatment	Positive, asymptomatic	Continue treatment.

In all cases, etiological treatment of Chagas disease should be accompanied by appropriate close follow-up, including liver enzymes and blood count parameters. See Echeverria et al. 2020 [68].

## Management of patients with Chagas cardiomyopathy and COVID-19

COVID-19 has been associated with multiple cardiac manifestations that include cardiac arrhythmias, Type 2 and 1 myocardial infarction, heart failure exacerbations and acute fulminant myocarditis [72,73]. Potential interactions between COVID-19 and CCC may be expected primarily due to the common immunological pathways shared by the diseases, as angiotensin-converting enzyme 2 (ACE2) is involved in heart function and the development of hypertension and diabetes mellitus, risk factors frequently observed in patients with CCC. ACE2 levels can be increased by the use of ACE inhibitors and/or angiotensin receptor blockers (ARBs) which are frequently used for the management of CCC. There is no evidence to date to support discontinuation of either ACE inhibitors or ARBs based on the theoretical potential of increasing the susceptibility to COVID-19 infection. Most cardiovascular societies including ESC, ACC, AHA, CCS, and the IASC have indicated that these medications should be continued regardless of the presence of concomitant COVID-19 manifestations. This should also be the case for patients with CCC.

Other potential interactions may occur in patients with CCC currently treated for cardiac arrhythmias, such as atrial fibrillation or life-threatening ventricular arrhythmias receiving amiodarone, as the potential for increased QT interval with treatments that have been proposed for COVID-19 such as hydroxychloroquine and/or azithromycin may increase the risk of torsade de points.

Patients with CCC must continue their usual treatments during the COVID-19 pandemic. Outpatient clinics may use telehealth, if available, to avoid putting these vulnerable patients at risk of SARS-CoV-2 infection. With proper hygiene and self-care measures, cardiac tests like EKG, echo, stress tests, or Holter may be performed or slightly delayed, always weighing risk-benefit and regional SARS-CoV-2 circulation status. If a patient with CCC develops new arrhythmias, stroke, or acute or worsening chronic heart failure, hospitalization must not be delayed. As hospitals in some regions may be severely strained by COVID-19, care of these acute events may be compromised, putting CCC patients at risk.

Table 2 lists potential interactions between cardiovascular drugs used to treat CCC and some proposed COVID-19 treatments.

**Table 2:** Potential interactions between COVID-19 treatments under investigation and CCM drugs.

COVID-19 treatments under investigation	Potential interactions with CCM drugs

Chloroquine-hydroxychloroquine	Inhibits CYP2D6 (increasing half-life of most of the beta blockers [74] and amiodarone), and inhibits and downregulates PgP [75]. They do not interact with novel oral anticoagulants (NOACS) or vitamin K antagonists (VKAs) [76].
Protease inhibitors (lopinavir-ritonavir)	By inhibiting CYP3A4, they increase plasma levels of most of CV drugs. May lower the effect of VKAs by induction of CYP2C19 and increase plasma levels of NOACs. Also may increase amiodarone levels [77].
Azithromycin	Increases levels of warfarin/acenocoumarol, these anticoagulants should be withdrawn during azithromycin treatment. Due to PgP inhibition, dose reduction of NOACs may be required.
Atazanavir	Increases levels of VKAs and NOACs (should be discontinued). May increase amiodarone levels and effect. May increase digoxin levels. Mild increase in atenolol levels (beta blocker) [77].
Remdesivir	No relevant interactions.
Favipiravir, Bevacizumab, Ecolizumab, Fingolimod, Pirfenidone, Interferon Methylprednisone	No relevant interactions.
Tocilizumab	May lower effect of anticoagulants.
Nitazoxanide	May increase VKA levels; do not use concomitantly.
Sarilumab	It is a CYP3A4 inducer, but dose modifications are not recommended.
Interferon and Methylprednisolone	Reduction of VKAs is advised.
Ribavirin	Interferes with the absorption of VKAs, possible dose increase indicated. Enalapril and other ACE2 inhibitors may provoke dry cough as well as ribavirin [78].
Ivermectin	May decrease the effect of warfarin and dicoumarol. Risk of myopathy with captopril [79].
Oseltamivir	No CYP interactions with CV drugs. However, case reports and series show some increase in the effect of VKAs [75].
Arbidol (Umifenovir)	May decrease metabolism of labetalol (beta-blocker) [80].
Canakinumab	No known drug interactions, but upregulation of CYP enzymes may further modify metabolization of CV drugs [81,82].
Anakinra	No drug interactions.
Emapalumab	No known drug interactions, but upregulation of CYP enzymes may further modify metabolization of CV drugs [83].
Siltuximab	VKA interaction through CYP3450. Close monitoring [84].
Cyclosporin A	Cyclosporin may increase digoxin levels. Amiodarone, losartan, and valsartan increase cyclosporin levels; ACE inhibitors increase nephrotoxicity [85,86].
Sirolimus	Serious warning; may increase risk of ACE inhibitor related angioedema. CYP450 and PgP interactions [87].
Darunavir/cobicistat	Drugs metabolized by CYP3A4, CYP2D6, or that use the transporters PgP, BCRP, MATE1, OATP1B1 or OATP1B3 may have interactions [88]. Anticoagulants, beta blockers, and digoxin should be used with caution.

### Future needs

Our current understanding of the potential interrelations between CD and COVID-19 is still limited; there are substantial needs for future research. The recommendations provided in this document should be considered preliminary and may require refinement and adjustment as our understanding of both diseases develops. Table 3 lists some of the most important gaps in our current clinical knowledge. Research from other disciplines will also be needed to better understand the epidemiology of both diseases, the social and psychological impacts of the pandemic on people with CD, new access barriers that emerge in the context of the pandemic and the economic dislocation it causes, and the particular contexts of vulnerable populations including migrants and indigenous communities.

**Table 3:** Understanding the interactions between COVID-19 and CD: Gaps and needs.

Disease interaction	Clinical questions	Drug development needs

How is the natural history of CD affected by COVID-19? Can the cytokine storm trigger reactivation of parasitemia? Does the cytokine storm trigger disease progression? Do viral and parasitic immune response pathways cross react? Does the chronic inflammatory state of CD lead to more severe COVID-19 disease? Does the prothrombotic state from both diseases behave synergistically?	What precautions are necessary regarding COVID-19 treatment in CD patients? What are the hemodynamic and arrhythmic risks of COVID-19 in patients with CCC? What is the impact of delaying CD treatments during COVID-19 infection? What is the impact of delays in access to CD diagnosis and cardiac evaluation? What is the impact of possible health system collapse on quality of care of CD patients with symptomatic disease?	What are the antiviral effects of antiparasitic drugs for CD (BZN and NFX)? Can anti-inflammatory drugs improve host response to COVID-19 and complement antiparasitic treatment of CD? Can allopurinol or colchicine help delay or avoid complications for both diseases? Is full anticoagulant therapy useful for COVID-19 [89] and CD [90]? Could CV CD treatments such as amiodarone treat COVID-19?

## Conclusion

While global in scope and indiscriminate in whom it infects, COVID-19 poses a particular risk to people with CD. Both diseases are more prevalent in marginalized populations, whose access to appropriate care is limited, and whose exposure to risk factors is proportionally higher. COVID-19 is more lethal in individuals with cardiac disease and/or other cardiac risk factors, such as diabetes and obesity, which are also prevalent in individuals with CD. The mechanisms of COVID-19 disease, while not completely understood, theoretically pose a risk of both exacerbation of cardiac dysfunction from CD and acute reactivation of CD due either to disease-induced immunomodulation or therapeutic immunosuppression. The economic impact of the pandemic hits hardest in the lowest socioeconomic strata, further complicating the ability of many individuals with CD to obtain the treatment they need for either illness. Efforts to mitigate the spread of COVID-19, by limiting medical facilities to all but the most urgent care, complicates efforts to diagnose, treat, and monitor patients with CD, which may lead to clinical deterioration, increased maternal-fetal transmission, and underdiagnosis, all of which were significant concerns even before the onset of the pandemic.

Roadblocks to accessing proper care for CD were recently described in the WHF-IASC Roadmap on CD [5]. Using a similar framework, Table 4 assesses the potential effect of the pandemic on key roadblocks to CD healthcare.

**Table 4:** Potential impact of COVID-19 on CD healthcare roadblocks.

Area	Potential impact of SARS-CoV-2 on key roadblocks

Prevention	– Reduced commitment from governments – Diversion of clinical research to COVID-19 – Public health resources diverted to COVID-19 – Lower media interest in neglected diseases – Limitations on health fairs, campaigns, and community events
Diagnosis	– Decreased visits to healthcare facilities out of fear of contagion – Testing/laboratory resources strained by COVID-19
Etiological treatment	– Decreased visits to healthcare facilities out of fear of contagion – Healthcare personnel strained by COVID-19 – Lack of knowledge on drug interactions with COVID-19, or with COVID-19 drugs
Diagnosis and treatment of clinical complications	– Limited knowledge of interaction between COVID-19 and CCC – Potential impact of COVID-19 drugs on CCC – Strains on health facilities’ ability to manage CCC
Psychosocial	– Increasing poverty due to economic impact of pandemic – Isolation from support networks – Fears about susceptibility to COVID-19 because of CD diagnosis

The end of the pandemic in Latin America is still far from sight and its full impact on healthcare and healthcare access, in particular, remains to be seen. CD has long been a hidden disease, with low awareness among healthcare professionals and people at risk, and limited commitment from governments. In the short-term, as public health resources are intently focused on mitigating the pandemic, it could become even more of a challenge to raise awareness of CD. At the same time, the pandemic could serve as an opportunity to strengthen public concern for addressing comorbid conditions and meeting the healthcare needs of underserved populations. Still, the current reality requires us to rethink traditional approaches to CD and other neglected diseases, to ensure we continue to make progress toward their eradication even as new public health challenges emerge. Ultimately, neither CD nor COVID-19 can be separated from their socioeconomic context, and winning the struggle against both diseases will involve implementing comprehensive programs that focus on strengthening the healthcare rights and access of the marginalized people who are currently most impacted.

## Footnotes

http://www.coalicionchagas.org/en_GB/news-article/-/asset_publisher/hJnt8AyJM2Af/content/preguntas-y-respuestas-sobre-covid-19-y-chagas).
